# Editorial: Unveiling the hidden arsenal: exploring secondary metabolites and fungal development in pathogenic fungi

**DOI:** 10.3389/fcimb.2025.1572135

**Published:** 2025-03-07

**Authors:** Kunlong Yang, Gang Ding

**Affiliations:** ^1^ Jiangsu Normal University-University of Wisconsin, Madison (JSNU-UWM) International Cooperation Joint Research Laboratory of Food Safety and Microbial Functional Genomics, School of Life Science, Jiangsu Normal University, Xuzhou, Jiangsu, China; ^2^ State Key Laboratory of Bioactive Substance and Function of Natural Medicines, Institute of Medicinal Plant Development, Chinese Academy of Medical Sciences and Peking Union Medical College, Beijing, China

**Keywords:** secondary metabolites, fungal pathogenicity, mycotoxins, antifungal strategies, fungal-host interactions

Pathogenic fungi pose a significant threat to global health and agriculture, contributing to a wide range of infections in both humans and plants. These organisms produce an array of secondary metabolites, which, while not essential for primary growth, play crucial roles in their adaptability, virulence, and interactions with host environments (Yang et al., 2022; Guo et al., 2023). The exploration of these metabolites has uncovered their diverse bioactivities, ranging from antimicrobial and immunosuppressive properties to their involvement in fungal morphogenesis and toxin production (Lara Da Costa et al., 2022). Understanding the biosynthesis, regulation, and functional roles of secondary metabolites in pathogenic fungi is essential for developing novel antifungal strategies and mitigating fungal diseases.

This Research Topic compiles cutting-edge studies that delve into the complex interplay between secondary metabolite biosynthesis, fungal development, and pathogenicity. The contributing articles offer a comprehensive examination of the molecular mechanisms governing secondary metabolite production, their influence on fungal virulence, and their interactions with host immune responses. Additionally, several contributions explore innovative methodologies for characterizing these metabolites and evaluating their potential as targets for antifungal therapies.

In this Research Topic, we highlight four pivotal studies addressing critical aspects in the field:

Jin et al., led by Prof. Jing Si from the Institute of Microbiology, Beijing Forestry University, renowned for advancing fungal genome annotation and natural product discovery, presented a high-quality genome assembly and annotation of *Sanghuangporus weigelae*. This medicinal fungus is traditionally used in East Asian pharmacopeia for its anti-inflammatory properties. By integrating multi-omics approaches, this study identified key metabolic pathways involved in the biosynthesis of bioactive compounds, such as terpenoids and polysaccharides, offering valuable insights into the medicinal applications of fungal secondary metabolites.

Zhao et al., from Institute of Quality Standard and Monitoring Technology for Agro-product (Guangdong Academy of Agricultural Sciences), specializing in mycotoxin risk assessment in food systems, revealed novel regulatory mechanisms of aflatoxin biosynthesis in *Aspergillus flavus*. Their work demonstrated that acetohydroxy acid synthase (AHAS) and dihydroxy acid dehydratase, key enzymes in branched-chain amino acid metabolism, directly modulated fungal growth, mycotoxin production, and pathogenicity. These findings provide actionable targets for disrupting toxigenic fungi in agricultural storage.

Ali et al., from Harbin Medical University, whose research focuses on clinical mycology and antifungal resistance epidemiology, featured research on the antifungal susceptibility of *Candida* species isolated from pregnant women in Yemen, shedding light on the increasing prevalence of *Candida* infections and the urgent need for effective antifungal treatments in resource-limited settings. The study underscores the growing concern of antifungal resistance and highlights the necessity for tailored therapeutic approaches.

Satterlee et al., from United States Department of Agriculture Toxicology and Mycotoxin Research Unit, pioneers in fungal chemical ecology, examined the chemical interactions between *A. flavus* and *Fusarium verticillioides*, two major maize pathogens. The findings reveal the competitive dynamics mediated by mycotoxins such as aflatoxin and fumonisin, offering new perspectives on fungal ecological interactions and potential strategies for controlling mycotoxin contamination in crops.

Collectively, these studies illuminate the dual nature of fungal secondary metabolites—as drivers of pathogenicity and reservoirs of therapeutic potential. They underscore the importance of integrating genomic, ecological, and clinical perspectives to combat fungal threats. As antimicrobial resistance escalates and climate change alters fungal biogeography, such interdisciplinary approaches will be pivotal in safeguarding global health and food security.

We hope that this Research Topic will catalyze innovation in antifungal discovery and inspire collaborative efforts to unravel the hidden complexities of fungal biology.
